# The DUF3715 domain has a conserved role in RNA-directed transposon silencing

**DOI:** 10.1261/rna.079693.123

**Published:** 2023-10

**Authors:** Theresa Schöpp, Daniil M. Prigozhin, Christopher Douse, Keisuke Kaji, Atlanta G. Cook, Dónal O'Carroll

**Affiliations:** 1 Centre for Regenerative Medicine, Institute for Regeneration and Repair, University of Edinburgh, Edinburgh EH16 4UU, United Kingdom; 2 Wellcome Centre for Cell Biology, University of Edinburgh, Edinburgh EH9 3BF, United Kingdom; 3 Berkeley Center for Structural Biology, Molecular Biophysics and Integrated Bioimaging Division, Lawrence Berkeley National Laboratory, Berkeley, California 94720, USA; 4 Lab of Epigenetics and Chromatin Dynamics, Department of Experimental Medical Science and Lund Stem Cell Center, Lund University, 221 84 Lund, Sweden

**Keywords:** transposon silencing, HUSH complex, piRNA pathway, DUF3715 domain and germline

## Abstract

RNA-directed transposon silencing operates in the mammalian soma and germline to safeguard genomic integrity. The piRNA pathway and the HUSH complex identify active transposons through recognition of their nascent transcripts, but mechanistic understanding of how these distinct pathways evolved is lacking. TASOR is an essential component of the HUSH complex. TASOR's DUF3715 domain adopts a pseudo-PARP structure and is required for transposon silencing in a manner independent of complex assembly. TEX15, an essential piRNA pathway factor, also contains the DUF3715 domain. Here, we show that TASOR's and TEX15's DUF3715 domain share extensive structural homology. We found that the DUF3715 domain arose in early eukaryotes and that in vertebrates it is restricted to TEX15, TASOR, and TASORB orthologs. While TASOR-like proteins are found throughout metazoa, TEX15 is vertebrate-specific. The branching of TEX15 and the TASOR-like DUF3715 domain likely occurred in early metazoan evolution. Remarkably, despite this vast evolutionary distance, the DUF3715 domain from divergent TEX15 sequences can functionally substitute the DUF3715 domain of TASOR and mediates transposon silencing. We have thus termed this domain of unknown function as the RNA-directed pseudo-PARP transposon silencing (RDTS) domain. In summary, we show an unexpected functional link between these critical transposon silencing pathways.

## INTRODUCTION

RNA-based surveillance mechanisms detect and silence young active transposons in both the mammalian soma and the germline. This is integral to the health of the organism and the survival of the species. Transposons have been very successful in colonizing genomes and their sequences or derivatives contribute to approximately half of the mammalian genomes (Lander et al. 2001; Mouse Genome Sequencing Consortium et al. 2002). The vast majority of transposons are dead, lacking the ability to transpose. Indeed, only LINE1 and the endogenous retrovirus (ERV) IAP elements have active copies in the mouse which can autonomously transpose (Naas et al. 1998; Goodier et al. 2001; Dewannieux et al. 2004), whereas the human genome has overcome ERVs and only active copies of LINE1 remain (Lander et al. 2001). While active transposon copies constitute for <1% of the total genome, they retain the potential to threaten genomic integrity. This is especially true in the germline where failure to silence transposons results in infertility (Bourc'his and Bestor 2004; Aravin et al. 2007; Carmell et al. 2007). Promoter DNA methylation is a potent mechanism of mammalian transposon repression (Walsh et al. 1998). However, there are periods in life where DNA methylation is reduced, absent or insufficient to mediate silencing (Greenberg and Bourc'his 2019). Active and evolutionary young transposons are silenced by the piRNA pathway in the germline (Molaro et al. 2014; Barau et al. 2016; Schöpp et al. 2020; Zoch et al. 2020) and the HUSH complex in the soma (Liu et al. 2018; Robbez-Masson et al. 2018).

The HUSH complex was identified in genetic screens for modifiers of transgene and transposon silencing (Tchasovnikarova et al. 2015; Liu et al. 2018; Robbez-Masson et al. 2018). TASOR, PPHLN1, and MPP8 were biochemically shown to comprise the core complex (Douse et al. 2020). Recent studies have shown that PPHLN1 is an RNA-binding protein (Prigozhin et al. 2020) that tethers the HUSH complex to the nascent transcript of target loci (Seczynska et al. 2022). TASOR acts as a scaffold and interacts with both PPHLN1 and MPP8, a chromatin-binding factor (Douse et al. 2020). The HUSH complex mediates chromatin-based transcriptional silencing through H3K9me3 and DNA compaction through SETDB1 and MORC2, respectively (Tchasovnikarova et al. 2015, 2017; Timms et al. 2016; Douse et al. 2018; Liu et al. 2018). The developing germline undergoes genome demethylation followed by de novo DNA methylation (Greenberg and Bourc'his 2019). It is the piRNA pathway that protects the integrity and continuity of the germline during this vulnerable period in development (Ozata et al. 2019). piRNAs are small RNAs, bound to PIWI proteins, guiding transposon silencing in the germline by multiple mechanisms (Ozata et al. 2019). In the cytoplasm, through base-complementarity piRNAs guide PIWI-mediated endonucleolytic cleavage of transposon transcripts. This event activates intricate piRNA biogenesis pathways that reinforce this cytoplasmic post-transcriptional silencing and also load the nuclear PIWI protein MIWI2 with a diverse repertoire of transposon-recognizing piRNAs (Ozata et al. 2019). Again, through base complementarity, piRNAs identify active transposon loci by binding their nascent transcripts; tethering of MIWI2 to the nascent RNA results in transcriptional silencing and DNA methylation (Ozata et al. 2019). The MIWI2-associated factors TEX15 and SPOCD1 are essential for these processes (Schöpp et al. 2020; Zoch et al. 2020). While not formally linked to the piRNA pathway, MORC1 is essential for the methylation of young, active transposons (Pastor et al. 2014).

While the HUSH and piRNA pathways appear distinct, they share some commonalities. Firstly, they both rely on transcription to identify the active transposons, with the nascent transcript serving as a platform where cotranscriptional silencing is initiated. Secondly, both systems utilize MORC proteins for transposon silencing (Pastor et al. 2014; Liu et al. 2018). Finally, TASOR and TEX15 share the DUF3715 protein domain (Schöpp et al. 2020). This domain is essential for TASOR function (Harten et al. 2014; Douse et al. 2020), it adopts a pseudo-PARP structure but has lost key residues that are required for enzymatic activity (Douse et al. 2020). The molecular function of the DUF3715 domain in TASOR is not understood but a role for complex assembly has been excluded (Douse et al. 2020). A function for TEX15's DUF3715 domain in transposon silencing remains unknown. Here, we explored the origin of the DUF3715 domain and a function for TEX15's DUF3715 domain in transposon silencing.

## RESULTS AND DISCUSSION

**FIGURE 1.**
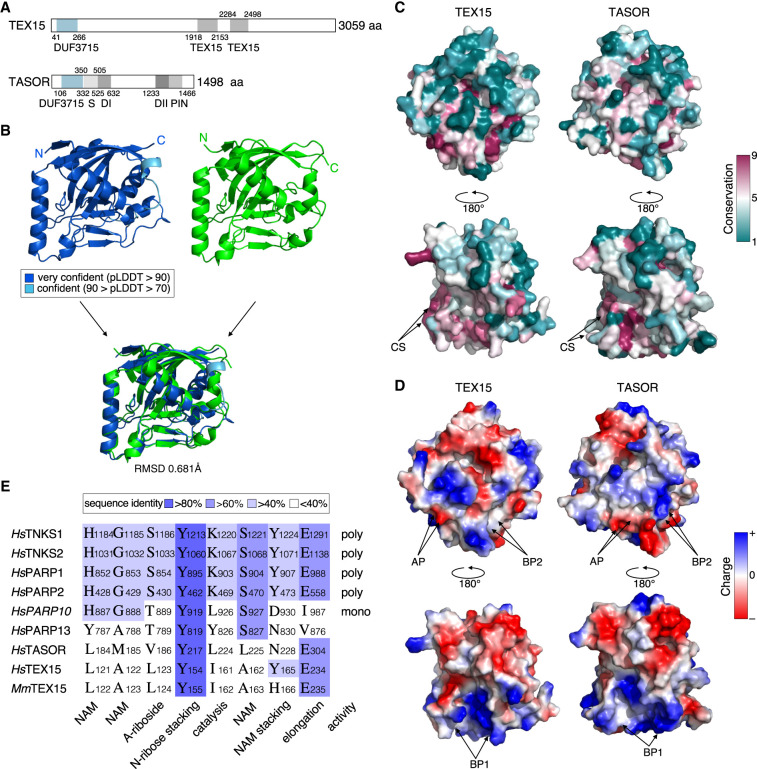
The DUF3715 domain of TASOR and TEX15 share extensive structural homology. (*A*) Schematic representation of TEX15 and TASOR domain structures. Characterized domains are in gray. (S) SPOC domain, (DI) DomI domain, (DII) DomII domain. (*B*) Structural prediction of TEX15 DUF3715 (colored by confidence [pLDDT] as indicated), experimental structure of TASOR DUF3715 (green) (PDB ID 6TL1 [Douse et al. 2020]) separately and aligned. (*C*) Surface views of TEX15 and TASOR as indicated from two different viewpoints via a 180° rotation. Color indicates the degree of conservation (magenta = conserved; teal = variable) across species. (CS) conserved patch. (*D*) Color indicates surface charge. ±64 ekT and ±66 ekT for TEX15 and TASOR, respectively. (AP) Acidic patch, (BP) basic patch. (*E*) Alignment of active PARP sites and NAD^+^ binding residues between PARP domain of selection of PARP family members and DUF3715 of TEX15 and TASOR. (NAM) Nicotinamide, (A-riboside) adenosyl-riboside, (N-riboside) nicotinamide-riboside. Sequence identity indicated in blue.

While TEX15 and TASOR both function in RNA-directed transposon silencing, their overall domain structure greatly differs (Fig. 1A). Both proteins share the DUF3715 domain (Fig. 1A). AlphaFold (Jumper et al. 2021; Tunyasuvunakool et al. 2021; Mirdita et al. 2022) models of TEX15's DUF3715 predict that it also adopts a pseudo-PARP structure, closely resembling TASOR's DUF3715 domain (Fig. 1B). While there are surface regions of high amino acid conservation (Fig. 1C), other features such as surface charge are more broadly shared between TASOR's and TEX15's DUF3715 domains (Fig. 1D). Residues required for PARP activity are poorly conserved in TEX15's DUF3715 domain as is the case for TASOR (Fig. 1E; Supplemental Fig. S1). In summary, the DUF3715 domain of TASOR and TEX15 share extensive structural homology.

**FIGURE 2.**
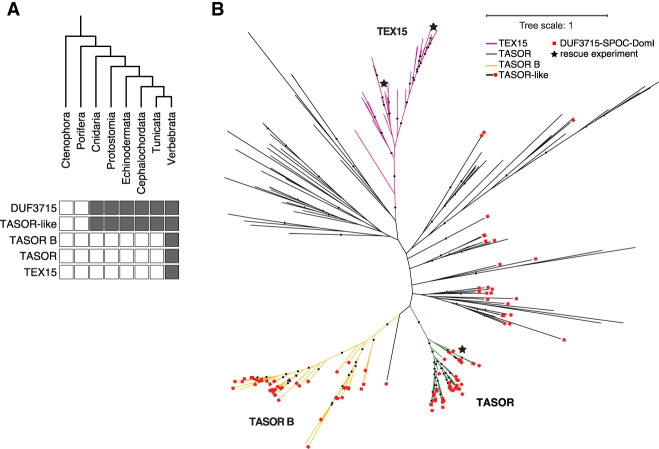
The DUF3715 domain arose in early eukaryotes and is restricted to orthologs of TEX15, TASOR, and TASORB in vertebrates. Phylogenetic analysis of DUF3715. (*A*) DUF3715-containing protein architectures are present across the metazoan tree of life, TASOR-like denotes architectures that contain DUF3715 followed by the SPOC–DomI domains as found in TASOR. (*B*) Unrooted maximum likelihood tree of DUF3715 sequences found across the tree of life. The vertebrate clades are shown in color, and invertebrate clades in black. The presence of the SPOC–DomI domains is indicated by red squares. Positions of human TASOR and human and zebrafish TEX15 DUF3715 domains used in the reconstitution experiment are indicated with black stars. Distance is average substitutions per site, bootstrap support over 80% is indicated as dots.

TEX15 domain architecture, comprising DUF3715 and one to two TEX15 domains, is found from fish to humans. The modern architecture of TASOR, including DUF3715, SPOC, DomI, DomII, and PIN domains (Fig. 1A), and TASORB is also found in vertebrates (Fig. 2A). The TASOR duplication that gave rise to TASOR and TASORB was likely an early vertebrate event (Fig. 2A,B). However, TASOR-like proteins defined by the presence of the amino-terminal DUF3715–SPOC–DomI domain combination are also present in invertebrates (Fig. 2A,B). Finally, the DUF3715 domain arose early in metazoan evolution and the branching of TEX15 and TASOR-like DUF3715 domains is also an ancient event (Fig. 2B).

**FIGURE 3.**
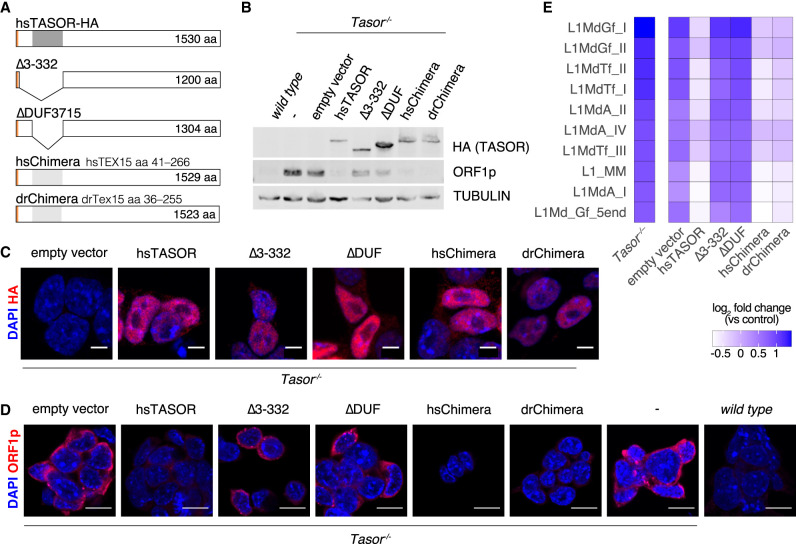
The TEX15 DUF3715 domain can functionally substitute TASOR's DUF3715 domain and mediate transposon silencing. (*A*) Schematic representation of human TASOR variants expressed in *Tasor*^−/−^ ESCs. All proteins contain an amino-terminal HA tag. (*B*) Representative image of western blot of mESC lysates from wild-type and *Tasor*^−/−^, with or without reconstitution as indicated (*n* = 2). (*C*,*D*) Representative immunofluorescence (IF) images of wild-type and *Tasor*^−/−^ with or without reconstitution as indicated (*n* = 2). DNA was stained with DAPI. (*C*) mESCs stained for HA (TASOR). Scale bars indicate 10 µm. (*D*) mESCs stained for LINE1 ORF1p. Scale bars indicate 5 µm. (*E*) Data were acquired by RNA-seq from mESCs. Comparison of transposon expression between control and *Tasor*^−/−^ with or without reconstitution as indicated (*n* = 2). Heat map shows the fold change of the 10 most up-regulated LINE1s in *Tasor*^−/−^ (*P* < 0.01, Benjamini–Hochberg adjusted two-sided Wald test).

It remains unknown if TEX15's DUF3715 domain is required for transposon silencing. The expression and function of TEX15 is restricted to the male germline (Yang et al. 2008, 2020; Schöpp et al. 2020) and thus it is challenging to perform structure–function analysis without the use of animal models. We therefore explored if the function of TEX15's DUF3715 domain could be tested in the context of TASOR. To this end, we generated *Tasor*-deficient mouse embryonic stem cell (ESC) lines using a genome editing approach (Supplemental Fig. S2). We identified two ESC lines that had homozygous loss-of-function alleles that resulted in the loss of TASOR protein and the deregulation of LINE1 silencing (Supplemental Fig. S2). We next generated a series of expression vectors encoding HA-tagged human TASOR variants that could be used to complement *Tasor*-deficient ESCs (Fig. 3A). In addition to wild-type TASOR, we made two DUF3715 deletion mutants of TASOR. The TASOR-Δ3-332 vector encodes amino-terminally truncated TASOR with a 329 amino acid deletion that encompasses the amino terminus and the DUF3715 domain (Fig. 3A; Douse et al. 2020). The TASOR-ΔDUF3715 domain construct expresses a TASOR protein with a clean deletion of the DUF3715 domain at amino acid 107–332 (Fig. 3A). Finally, we generated two chimeric TASOR proteins where the DUF3715 domain is replaced with that of human TEX15 (TASOR_hsTEX15-DUF3715) or zebrafish TEX15 (TASOR_drTEX15DUF3715) (Fig. 3A). The above constructs were stably integrated into *Tasor*-deficient ESC lines and expression of the respective proteins was achieved (Fig. 3B). Furthermore, each of these TASOR variants localized to the nucleus (Fig. 3C). Wild-type TASOR but not the DUF3715-deficient variants could restore LINE1 silencing (Fig. 3B,D,E). Strikingly, the expression of the chimeric TASOR_hsTEX15-DUF3715 and TASOR_drTEX15DUF3715 proteins could also mediate LINE1 silencing in *Tasor*-deficient ESCs (Fig. 3B,D,E).

The comparison of the structure of the TASOR DUF3715 domain (Douse et al. 2020) with the AlphaFold model (Jumper et al. 2021; Tunyasuvunakool et al. 2021) of the TEX15 DUF3715 domain revealed extensive structural homology between the two distantly related domains that also extends to surface charge conservation (Fig. 1D). Despite this structural homology, it cannot be assumed that the TEX15's DUF3715 domain has a direct role in transposon silencing. Here, we irrefutably show that the human TEX15 DUF3715 domain can mediate transposon silencing in the context of TASOR. Furthermore, this ability is a conserved feature of vertebrate TEX15 DUF3715 domains given the zebrafish domain is also functionally proficient within TASOR in ESCs. These feats are remarkable given that the bifurcation of TEX15 and TASOR-like DUF3715 domains is an ancient event occurring in a common vertebrate precursor (Fig. 2A). The molecular function of both TASOR and TEX15 DUF3715 domains remains unknown (Douse et al. 2020; Schöpp et al. 2020; Yang et al. 2020) but we have demonstrated an essential role for them in transposon silencing. Having revealed a conserved role for this “domain of unknown function” in RNA-directed transposon silencing, we have termed it the RNA-directed pseudo-PARP transposon silencing (RDTS) domain. Mutations in human TEX15 are associated with male infertility (Okutman et al. 2015; Colombo et al. 2017). Should disease-associated variants be found within TEX15's RDTS domain, the genetic reconstitution assay presented in this paper could be used to test the functionality of these mutants and has the potential to define them as disease-causing or bone fide pathological variants. TEX15 is essential for piRNA-directed transposon silencing and methylation in the male mouse germline (Schöpp et al. 2020; Yang et al. 2020). *Tasor*-deficient mice die early during development due to gastrulation failure (Harten et al. 2014). In mammals, the germline is an acquired lineage and it is formed from epiblast-derived cells early during embryonic development (Ohinata et al. 2005; Vincent et al. 2005). Thus, the RDTS domains of both, TASOR and TEX15, are critical in safeguarding the genomic integrity of the immortal lineage and the continuity of life.

## MATERIALS AND METHODS

### Cell lines and maintenance

E14Tg2a mESCs were used in this study. Cells were maintained in LIF/FCS mESC media (GMEM (G5154, Sigma-Aldrich), 10% FCS (10270106, Life Technologies), 0.1 mM 2-mercaptoethanol (31350010, Gibco), 2 mM l-glutamine (25030-024, Gibco), 1 mM sodium pyruvate (11360070, Gibco), 1× nonessential amino acids (11140035, Gibco), 1× penicillin–streptavidin (both final concentration of 100 U mL^−1^, 15140122, Gibco), 55,000 units Leukemia inhibitory factor (LIF) (CRM TC facility) on plates coated with 0.1% gelatin (G1890, Sigma-Aldrich).

### Generation of *Tasor*-deficient ESC lines

The *Tasor*-null allele was generated using CRISPR–Cas9 gene-editing technology with a single sgRNA as described (Ran et al. 2013). Therefore, sgR1 5′-GGTATCCTCGGTCTCCTAA-3′ was cloned into CAS9 encoding pX549_Cas9_2A_Pu. 2 × 10^6^ cells were nucleofected with Mouse ES Cell Nucleofector Kit (VAPH1001, Lonza) following the manufacturer's recommendation with small changes. In brief, 90 µL nucleofector solution and 20 µL Supplement 1 were mixed with 2 µg cDNA. Cells were resuspended in the mix, transferred into a cuvette and nucleofected using a nucleofector device 2b set to A-023. Cells were then transferred into 10 mL warm media and plated on gelatin-coated plates. After 24 h, the media was changed to selection media (mESC media supplemented with 1 µg mL^−1^ puromycin [P8833, Sigma-Aldrich]) for 48 h. Media was then changed back to mESC media and cultures were maintained for 7 d or until colonies were visible. Single colonies were picked and transferred into a 96-well plate and expanded in 48-well plates until sufficient material was available for genotyping.

### DNA isolation and genotyping

For genotype analysis, cells were pelleted and resuspended in lysis buffer (0.45% NP-40 (NP40S, Sigma-Aldrich), 0.45% Tween-20 (P9416, Sigma-Aldrich), 0.2 mg mL^−1^ Proteinase K, 1× DreamTaq PCR buffer in water). Volume was adapted to cell amount, for one-quarter of a 96-well plate, 30–50 µL were used. For PCR, 1 µL of DNA containing lysate, 1× DreamTaq Green buffer, 0.2 mM dNTPs, 1 µM primer mix (Ex12F2 5′-CAGCATACTGCCTTGCAAATAA-3′, Ex12R2 5′-TGATTCCACAAAAATAATCCCAG-3′) and Taq polymerase were mixed and brought to 15 µL with H_2_O. Clones were screened for *Tasor*-null alleles using Sanger sequencing. Results were analyzed with TIDE (Brinkman et al. 2014).

### Genetic reconstitution of *Tasor*-deficient ESCs

*Tasor*-deficient mESC lines were reconstituted by nucleofection (as described above) using the *PiggyBac* (PB) system (Wang et al. 2008; Yusa et al. 2011) with 1 µg of pBase (transposase) and 1 µg of either PB-CAG-iGFP-MCS, PB-CAG-iGFP-hsTasor, PB-CAG-iGFP-hsTasor-Δ3-332, PB-CAG-iGFP-hsTasor-ΔDUF, PB-CAG-iGFP-hsTasor-hsTex15-DUF-Chimera, or PB-CAG-iGFP-hsTasor-drTex15-DUF-Chimera. Cells were then transferred into medium and plated on gelatin-coated plates. Three to four days post nucleofection GFP^+^ cells were sorted with a FACS Aria II or Fusion (BD) as described below.

### FACS sorting of ESCs

For sorting GFP^+^ ESCs, cells were dissociated from plates using Accutase (A1110501, Gibco) for 3–5 min at 37°C. Accutase was then diluted with mES media and cells pelleted for 5 min at 300 rcf and resuspended in PBS with 2% FCS. An amount of 1 µg mL^−1^ DAPI was added to the cell suspension and GFP^+^ cells were sorted on a BD Fusion or Aria II into mES media at room temperature (Supplemental Fig. S3). After the sort cells were pelleted for 5 min at 300 rcf, resuspended in fresh media and plated on 0.1% gelatin-coated plates. GFP^+^ cells were at least sorted twice. The first sort was typically performed 3–4 d post nucleofection and the second one ∼2 wk post nucleofection. The gating strategy for the GFP^+^ population used is shown in Supplemental Figure S3.

### Antibodies

The following antibodies were used in the study: anti-HA (C29F4, Cell Signaling Technologies, Lot#9 and Lot#10, IF: 1:1000 [HA-TASOR]; WB: 1:1000 [HA-TASOR]); anti-LINE1-ORF1p (Di Giacomo et al. 2013) (IF: 1:500); WB: 1:500, anti-rabbit (Alexa Fluor 488, 568, 647, Cat#A-21206, A10042, A-31573, IF 1:1000); anti-FAM208A/TASOR (HPA006735, Atlas Antibodies, WB 1:500) and anti-αTubulin (T9026, Merck, WB 1:500).

The anti-HA antibody was validated for IF against mouse samples containing no HA epitope-tagged proteins (Schöpp et al. 2020) as done previously for western blotting (WB) (Zoch et al. 2020). The anti-LINE1-ORF1p (described previously, Di Giacomo et al. 2013) antibody has been previously validated for IF on mouse sections with and without the according protein present and was used in several studies since. The anti-TASOR antibody was tested for WB in previous studies (Tchasovnikarova et al. 2017; Douse et al. 2020) as well as this study and validated on lysates with and without TASOR.

### Western blotting

Cultured cells were lysed post trypsinization in mild lysis buffer (100 mM KCl, 2.5 mM MgCl_2_, 50 mM Tris-HCl pH 8, 0.1% Triton X-100) on ice for 20 min. mESCs were additionally sonicated with 4× 10 sec on, 30 sec off using a Bioruptor Pico (Diagenode). Cell lysates were cleared by centrifuging for 5 min at 21,000 rcf. The proteins contained in the supernatant were separated on 4%–12% Bis-Tris polyacrylamide gels (NuPAGE Mini, Invitrogen) according to the manufacturer's instructions. Proteins were transferred onto a 0.45 µm nitrocellulose membrane (Amer sham Protran; 10600007, GE Healthcare), blocked in 3.5% skimmed milk in TBS-T (1× TBS, 0.1% Tween 20 [P1379, Sigma-Aldrich]) and stained with primary antibody diluted in blocking solution and incubated at 4°C overnight or 1 h at room temperature (anti-HA C29F4 was used 1:1000, anti-LINE1ORF1p 1:500, anti-TASOR HPA006735 1:500, and anti-αTubulin 1:500), washed three times in TBS-T and incubated with LI-COR fluorescent-conjugated secondary antibodies (anti-rabbit IRDye 800CW [926-32213, LI-COR Biosciences], anti-mouse IRDye 800CW [926-32212, LI-COR Biosciences], anti-rabbit IRDye 680RD [926-68073, LICOR Biosciences]) diluted 1:10,000 in TBS-T. Images were acquired and analyzed using a LI-COR Odyssey Imager and Image Studio Lite (version 5.2.5).

### Immunofluorescence

mESCs were passaged and a small number of cells plated on gelatin-coated Ibidi imaging chambers (IB-80841, Thistle Scientific) and grown until the desired density was reached. All media was aspirated and cells washed twice with cold PBS, followed by 4% PFA (15512, Sigma-Aldrich) fixation for 10 min. Cells were again washed with PBS, permeabilized using 0.3% Triton in PBS, and blocked for 1 h at room temperature in blocking solution (10% natural donkey serum [D9663, Merck], 1% bovine serum albumin [BSA] [B6917, Sigma-Aldrich], 0.1% glycine [Sigma-Aldrich] in PBS). Primary antibodies were diluted in blocking buffer and incubated at 4°C overnight (anti-HA [C29F4, Cell Signaling Technologies] 1:500, anti-LINE1-ORF1p [Di Giacomo et al. 2013] 1:500). Cells were then washed 3× with PBS before incubation with Alexa Fluor secondary antibodies (donkey anti-rabbit or donkey anti-mouse 488, 568, or 647) for 1 h at room temperature, again washed 2× with PBS and mounted using Prolong Gold (P36930, Invitrogen) and let to dry overnight. DAPI was added 1:1000 (5 µg mL^−1^) to the secondary antibody mix as counterstain, all antibodies were diluted in blocking solution.

Images were acquired on a Zeiss LSM880 with Airyscan module. When acquired, the Airyscan module was used and images were deconvoluted using “Airyscan processing” in the Zeiss Zen software set to “3D” and strength 6. Images were then processed and analyzed with ImageJ (v2.0.0-rc-65/1.51u).

### RNA sequencing (RNA-seq) and analysis

For RNA-seq from mESCs, total RNA was extracted from one well of a six-well plate of 60%–80% confluent cells with QIAzol reagent following the manufacturer's recommendation. Libraries were prepared with NEBNext Ultra II Directional RNA Library Kit for Illumina with prior use of NEBNext rRNA Depletion Kit v2 (E7405, NEB) following the manufacturer's protocol. Libraries were amplified using eight PCR cycles and sequenced on a NextSeq 500 (Illumina) in 75 bp single-end read mode.

For downstream analyses, adaptor sequences were removed from the reads with cutadapt (Kechin et al. 2017) (1.18) using default settings. For the analysis of differentially expressed retrotransposons, consensus sequences of rodent retrotransposons were retrieved from Repbase (24.01) and used to map the processed reads using bowtie2 (Langmead and Salzberg 2012) (2.4.2) with default settings. The number of mapped reads per retrotransposon were counted and analyzed using DESeq2 (1.32.0) (Love et al. 2014).

### Phylogenetic analysis

Because the DUF3715 hidden Markov model (HMM) contained in the Pfam database (Mistry et al. 2021) does not cover the whole experimental structure of TASOR DUF3715, a longer HMM for the DUF3715 domain was constructed as follows. Protein sequences were gathered by BLAST searching the NR protein sequence database (Sayers et al. 2022) with a query corresponding to the solved crystal structure of TASOR DUF3715 (residues 111–328). Results were truncated at 95% coverage, 25% sequence identity, realigned with MAFFT (Katoh and Standley 2013), and HMM was constructed using the hmmbuild command from the HMMER suite version 3.3.2 (Eddy 2011). HMM for SPOC–DomI was built by phmmer searching the UniProt database (UniProt Consortium 2021) with a query corresponding to the AlphaFold-predicted TASOR SPOC–DomI (residues 354–633). Hits covering <70% of the query sequence were excluded, and HMM was constructed using the hmmbuild command from the HMMER suite.

To identify sequences containing the DUF3715 domain throughout the tree of life, we used hmmsearch against the UniRef50 sequence database (Suzek et al. 2015). We then matched obtained sequences to all HMM models in the Pfam database (Mistry et al. 2021) to remove hits that better matched other domain definitions. This was done to prevent potential low scoring hits corresponding to actual PARP domains from being included in further analysis. The 239 resulting sequences were realigned to the extended DUF3715 model using hmmalign. Maximum likelihood phylogeny was constructed using RAxML version 8.2.12 (Stamatakis 2014) with 100 bootstrap replicates (raxmlHPC-PTHREADS-AVX -T 8 -f a -x 12345 -p 12345 -# 100 -m PROTCATJTT). We used Taxoniq (taxoniq.github.io) to determine the class and phylum distribution of identified DUF3715-containing sequences from their species names. The tree was visualized, and figures prepared in iToL (Letunic and Bork 2021).

### Alignments

Protein multiple sequence alignments were generated using Clustal Omega (Madeira et al. 2019) and presented using Jalview (Waterhouse et al. 2009) with color indicating “percentage identity.” The following sequences were used in the alignments: NP_001337091.1 (TEX15 Homo sapiens), XP_006509040.1 (TEX15 Mus musculus), NP_001352564.1 (TASOR Homo sapiens), NP_001609.2 (PARP1 Homo sapiens), NP_001036083.1 (PARP2 Homo sapiens), NP_116178.2 (PARP10 Homo sapiens), NP_001350420.1 (PARP13 Homo sapiens), NP_003738.2 (TNKS1 Homo sapiens), NP_079511.1 (TNKS2 Homo sapiens).

### Protein structure modeling and analysis

The model of N-terminal human TEX15 (NP_001337091.1) was generated by AlpaFold2 (Jumper et al. 2021; Tunyasuvunakool et al. 2021; Mirdita et al. 2022) using MMSeqs2 (https://colab.research.google.com/github/sokrypton/ColabFold/blob/v1.3.0/AlphaFold2.ipynb). PyMOL (2.3.3) was used to visualize the generated models and to calculate the electrostatic surface charge. Alignment between TEX15 DUF3715 and TASOR DUF3715 was performed using PyMOL “align” allowing refinement (displayed RMSD value corresponds to 141–141 atoms). Surface conservation analysis was performed using ConSurf (Ashkenazy et al. 2016). Multiple sequence alignment generated with automatic homologs selection by ConSurf using default settings (HMMER, 1 iteration, 0.0001 *E*-value cutoff and database UNIREF-90).

### Statistical information

Statistical testing was performed with R (4.1.0) using the RStudio software. Unpaired, two-sided Student's *t*-tests were used to compare differences between groups and Wald tests and Benjamini–Hochberg correction were used for RNA-seq data analysis. Averaged data are presented as mean ± SEM (standard error of the mean) unless otherwise indicated. No statistical methods were used to predetermine sample size. The experiments were not randomized, and the investigators were not blinded to allocation during experiments and outcome assessment.

## DATA DEPOSITION

The RNA-seq data generated in this study have been deposited at Gene Expression Omnibus under accession number GSE234730.

## SUPPLEMENTAL MATERIAL

Supplemental material is available for this article.

## Figures and Tables

**Theresa Schöpp:**
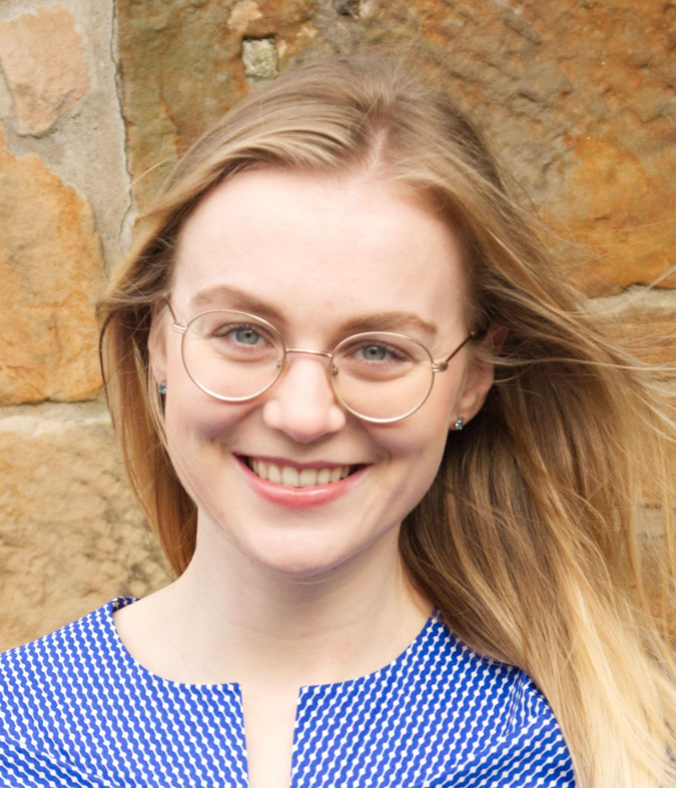


## MEET THE FIRST AUTHOR

***Meet the First Author(s)* is an editorial feature within *RNA*, in which the first author(s) of research-based papers in each issue have the opportunity to introduce themselves and their work to readers of *RNA* and the RNA research community. Theresa Schöpp is the first author of this paper, “The DUF3715 domain has a conserved role in RNA-directed transposon silencing.” She completed her PhD in the O'Carroll laboratory at the University of Edinburgh where she studied the nuclear mechanism of piRNA guided transposon silencing in the developing mammalian germline.**

**What are the major results described in your paper and how do they impact this branch of the field?**

We connect two distinct RNA-directed transposon silencing mechanisms, namely the piRNA pathway and the HUSH complex, by in-depth investigation of a shared domain of unknown function within the proteins TEX15 and TASOR, essential factors to the respective pathways. Although these domains separated in early metazoa, the expression of TASOR where its DUF3715 domain was swapped with the one of human or zebrafish TEX15 reestablished transposon silencing in mutant mouse ES cells.

**What led you to study RNA or this aspect of RNA science?**

When I decided to pursue a PhD, I was eager to learn more about the specificity of transcription and epigenetics; this introduced me to the piRNA pathway—I had never before heard about this type of small RNAs but was immediately fascinated by their particularities. The fact that they act in a highly specific but adaptable manner and function as our genome's immune system still excites me.

**During the course of these experiments, were there any surprising results or particular difficulties that altered your thinking and subsequent focus?**

The experiments presented in this paper were the result of the struggle I had when studying TEX15 in the developing germline—a large protein that presented various difficulties. We knew it had three conserved domains, and I was intrigued to understand what they were doing when I found that TASOR possessed the same domain of unknown function. Reading up on the HUSH complex gave me this subtle feeling that maybe there could be some similarities in their function. Poor success in expressing TEX15 in vitro then finally led to the idea of a swap experiment; that it simply worked was probably the biggest surprise.

**What are some of the landmark moments that provoked your interest in science or your development as a scientist?**

I was a very curious child who has always loved the outdoors; however, at some point I realized that I barely understood anything of what was going on around me in detail. Still in high school and full of questions, I audited a lecture on neurobiology. I was completely blown away—it sparked my scientific curiosity and I knew I had to study molecular biology. I was also lucky to meet inspiring people along the way who made me believe that I couldn't just learn about science, but contribute to it.

**If you were able to give one piece of advice to your younger self, what would that be?**

Sometimes things simply won't work out the way you planned, but don't let that drag you down. Instead, take a few steps back, think what this might tell you, look at it from a different angle and start fresh.

**Are there specific individuals or groups who have influenced your philosophy or approach to science?**

It is quite a list if I think back, but most influential was definitely my time in Edinburgh. There was no hierarchy among our group and everyone wanted to learn from one another and together. This and my supervisor's approach of managing a laboratory made me see that research isn't only about experiments but is also about a great team.

**What are your subsequent near- or long-term career plans?**

After finishing my PhD, I decided to leave academia and pursue a career in industry. It was a tough decision, but also time for something new.
